# Simple method for evaluating achievement degree of lung dose optimization in individual patients with locally advanced non‐small cell lung cancer treated with intensity modulated radiotherapy

**DOI:** 10.1111/1759-7714.14634

**Published:** 2022-08-31

**Authors:** Takanori Abe, Misaki Iino, Satoshi Saito, Tomomi Aoshika, Yasuhiro Ryuno, Tomohiro Ohta, Mitsunobu Igari, Ryuta Hirai, Yu Kumazaki, Yu Miura, Kyoichi Kaira, Hiroshi Kagamu, Shinei Noda, Shingo Kato

**Affiliations:** ^1^ Department of Radiation Oncology, International Medical Center Saitama Medical University Saitama Japan; ^2^ Department of Respiratory Medicine, International Medical Center Saitama Medical University Saitama Japan

**Keywords:** intensity modulated radiotherapy, locally‐advanced lung cancer, lung dose optimization

## Abstract

**Background:**

In this study, we developed a simple method for evaluating achievement degree of lung dose optimization in individual patients with locally advanced non‐small cell lung cancer (NSCLC) treated with intensity modulated radiotherapy (IMRT).

**Methods:**

Data of 28 patients with stage IIB to IIIC NSCLC were retrospectively analyzed. All patients were treated with IMRT and a simulated three‐dimensional conformal radiotherapy (3D‐CRT) plan created for them. Dose‐volume parameters of lung were analyzed for their correlation with radiation pneumonitis (RP).

**Results:**

Over a median follow‐up of 14 months, grade 1 pneumonitis was diagnosed in 14 patients (50%), grade 2 pneumonitis in 11 (39%), and grade 3 pneumonitis in one (4%). Two patients did not develop pneumonitis. None of the patients developed grade 4 or 5 pneumonitis. Regarding dose‐volume parameter ratios between IMRT and simulated 3D‐CRT, receiver operating characteristic analysis showed that mean lung dose (MLD)_IMRT_/MLD_3D‐CRT_ had the largest area under curve (0.750). Cumulative 6‐month incidences of grade 2 or greater RP were 78.4% versus 19.5% (MLD_IMRT_/MLD_3D‐CRT,_ ≥1.0 or less); this difference was significant (*p* < 0.05).

**Conclusions:**

We found that cutoff values for dose volume parameter ratios significantly predict grade 2 or greater RP. We believe that these parameter ratios could be useful in assisting evaluation of achievement degree of lung dose optimization in IMRT for LA‐NSCLC.

## Graphical abstract

Incidence of grade 2 or greater pneumonitis between two groups divided by MLDIMRT/MLD3D‐CRT ≥1.0 or less.

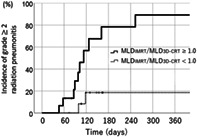

## INTRODUCTION

Concurrent chemoradiotherapy (CCRT) is a standard form of curative treatment for locally advanced non‐small cell lung cancer (LA‐NSCLC).^1^ The PACIFIC trial recently reported that consolidative therapy with durvalumab after CCRT significantly prolongs overall and progression‐free survival.^2^ Another recent change in treatment of LA‐NSCLC has been improvement in radiotherapy techniques. Intensity‐modulated radiotherapy (IMRT) is a means of concentrating the radiation dose on the target and sparing surrounding normal tissue.^3^ In patients with LA‐NSCLC, IMRT enables delivery of significantly lower doses to the lung, heart, and esophagus than is achieved with conventional three‐dimensional conformal radiotherapy (3D‐CRT).^4^ There have been no direct comparisons between IMRT and 3D‐CRT; however, several retrospective studies have found that IMRT led to reduced toxicity while equaling or improving survival outcomes.^5^, ^6^ Therefore, IMRT has been increasingly administered to patients with LA‐NSCLC, especially in the Western world during the last decade.^7^ Although IMRT for LA‐NSCLC is being increasingly used worldwide,^7^, ^8^, ^9^, ^10^, ^11^ the proportion of the facilities where IMRT for LA‐NSCLC is performed varies widely in each region, mainly due to limited human and machine resources.^12^ One of the obstacles in performing IMRT for LA‐NSCLC is the difficulty in the treatment plan optimization process because it is more complex than for 3D‐CRT. With 3D‐CRT, lung dose distribution may not vary much once target volume is determined. However, with IMRT to the same target volume, lung dose optimization can vary widely because dose distribution can easily be modified by adjusting optimization settings. Additionally, some researchers have expressed concern that the incidence of radiation pneumonitis (RP) may be slightly higher in specific ethnic groups after CCRT followed by durvalumab.^9^, ^10^, ^11^, ^12^, ^13^, ^14^, ^15^, ^16^ Thus, it is important to create safe and reproducible IMRT plans for treating LA‐NSCLC. However, in the absence of established means of determining clinical goals for lung dose optimization in individual patients, it is difficult to be sure that adequate optimization has been achieved or not. In this study, we developed simple methods for evaluating achievement degree of lung dose optimization in individual patients and evaluated its utility with an endpoint RP.

## METHODS

### Patients

Relevant data of patients with LA‐NSCLC who had been treated with CCRT using IMRT followed by durvalumab in our institution between March 2020 and August 2021 were retrospectively analyzed. Histological diagnoses had been made by examining bronchoscopy biopsies from all patients. Additionally, all patients had undergone computed tomography (CT), magnetic resonance imaging of the brain, and fluoro‐deoxy‐glucose positron emission tomography. Tumors were classified in accordance with the TNM classification of malignant tumors (eighth edition). Treatment modalities such as radiotherapy or surgery were carefully chosen by our hospital's tumor board, which consists of a thoracic surgeon, thoracic medical oncologist, radiologist, and radiation oncologist. This study was approved by our Institutional Review Board (reference number: 20–167) and performed in accordance with the Helsinki Declaration of the World Medical Association. Written informed consent for the use of medical data was obtained from all study patients.

### Radiotherapy

**FIGURE 1:**
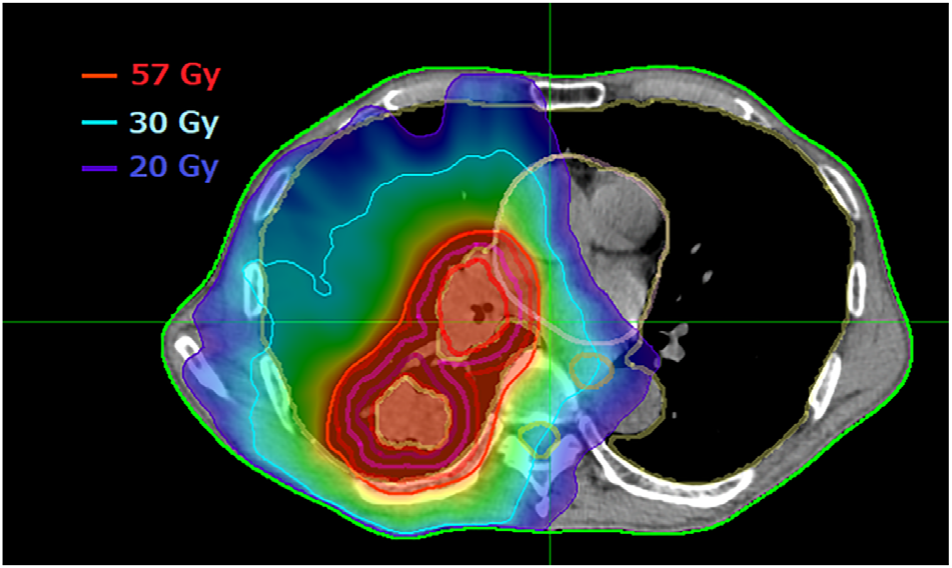
Representative actual treatment plan of intensity modulated radiotherapy (volumetric modulated arc radiotherapy). Two arcs were used with arc angles of 10–181 degrees and 181–10 degrees, respectively

IMRT was performed using a volumetric modulated arc radiotherapy (VMAT) technique in all the patients. A representative image of the VMAT plan is shown in Figure 1. Four‐dimensional CT and CT in the mid‐respiratory phase were performed for treatment planning. The thickness of CT in the mid‐respiratory phase was 2.5 mm. Doses were calculated on the basis of CT findings in the mid‐respiratory phase and four‐dimensional CT was used to create the internal gross tumor volume (IGTV) with maximum intensity projection methods. A clinical target volume (CTV) margin of 5 mm was added to the IGTV. Bony structures and other organs with no evidence of invasion by tumor were excluded from the CTV. In this study, the elective nodal area was not included in the CTV. A planning target volume (PTV) margin of 5 mm was added to the CTV in all directions. A total of 60 Gy in 30 fractions was prescribed to cover 95% of the PTV. Dose constraints for organs at risk were as follows: less than 35% of lung volume to receive more than 20 Gy (lung V_20_), less than 60% of lung volume to receive more than 5 Gy (lung V_5_), maximum dose (Dmax) to spinal cord less than 50 Gy, less than 25% of esophageal volume to receive more than 40 Gy, and less than 20% of heart volume to receive more than 40 Gy. To calculate lung dose, GTV was excluded from lung. Treatment plans were created using Eclipse (Armonk), the dose calculation algorithm being AAA.

### Chemotherapy

The general conditions of patients were carefully checked by thoracic medical oncologists to determine whether chemotherapy was indicated. Generally, patients with ECOG performance status of 0–2 and good organ function were considered candidates for CCRT. Interstitial pneumonia is considered a contraindication to CCRT in our hospital. Individual chemotherapy regimens were determined by medical oncologists.

### Durvalumab

After CCRT, all patients received durvalumab biweekly until development of grade 2 or greater toxicity, or completion of 24 cycles. Toxicities were classified in accordance with the National Cancer Institute Common Toxicity Criteria for Adverse Events, version 5.0. Blood tests and chest radiographs were routinely performed at every hospital attendance for administration of durvalumab. If pneumonitis or disease progression was suspected, chest CT and any other examinations deemed necessary were performed.

### Dose volume parameters of radiotherapy

In addition to lung V20 and lung V5, mean lung dose (MLD), and volumes of lung receiving more than 30 Gy (lung V30), more than 40 Gy (lung V40), more than 50 Gy (lung V50), and more than 60 Gy (lung V60) were calculated and recorded.

### Simulated 3D‐CRT plan

**FIGURE 2:**
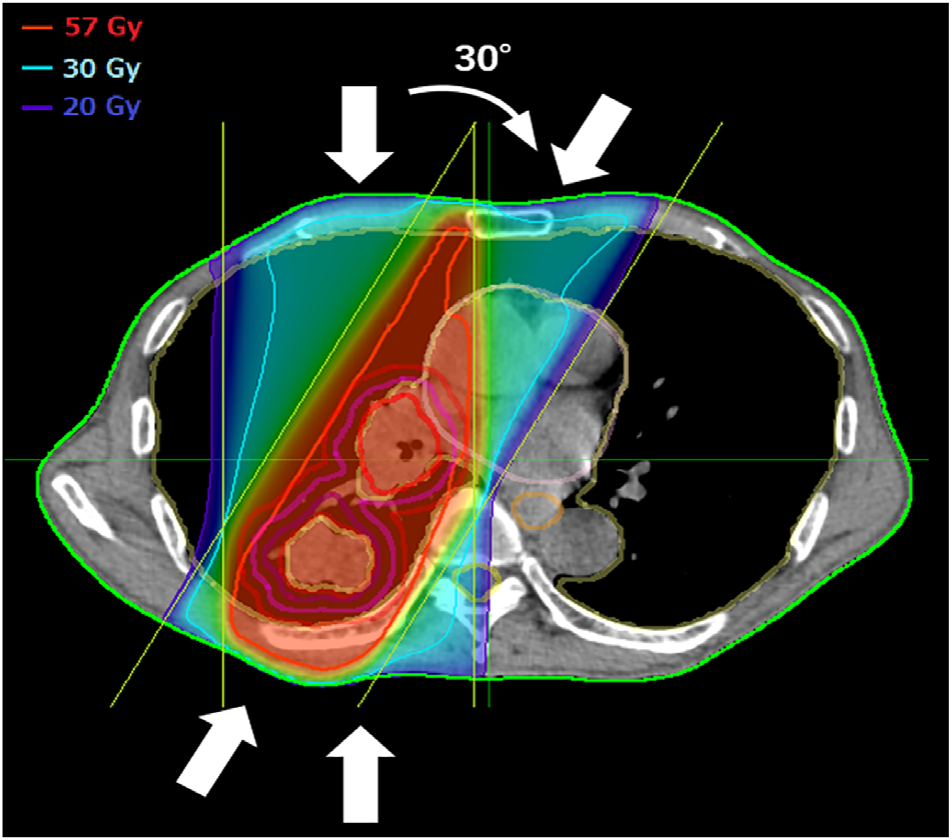
Representative treatment plan of simulated 3D‐CRT as same patient in Figure 1. The thick white arrow indicates the beams. There are four beams in the anterior‐posterior direction and 30–210 degrees in diagonal directions

For a reference, 3D‐CRT plans were created on CT images for IMRT with the same target volume. Anterior‐posterior beams and 30°of diagonal beams were used. A representative image is shown in Figure 2. The weights of anterior‐posterior:diagonal beams were set at 2:1. Sixty Gy in 30 fractions was prescribed to the isocenters of the beams, which were set in the centers of the PTV and adjusted slightly when they were on air in lung parenchyma. To create the irradiation field, a multileaf collimator (MLC) margin of 5 mm was added to the PTV and beam angle or shape of MLC was not modified to spare the spinal cord because this simulated plan was only for reference. The treatment planning system and dose calculation algorithm were the same as for IMRT. The same dose volume parameters for lung as with IMRT were calculated and recorded. To evaluate differences between the IMRT and simulated 3D‐CRT plans, the ratios of dose‐volume parameters such as V5_IMRT_/V5_3D‐CRT_, V20_IMRT_/V20 _3D‐CRT,_ and MLD_IMRT_/MLD_3D‐CRT_ were calculated.

### Statistical analysis

The incidences of grade 2 or greater RP were compared between the two groups with the Kaplan–Meier method and log–rank test. Cutoff values for creating the two groups were determined by receiver operating characteristic (ROC) analysis. *p* < 0.05 was considered to denote significant differences and all tests were two‐sided. All statistical analyses were performed using IBM SPSS Statistics for Windows, version 25.0 (IBM Corp.). Scatter diagrams were created using Excel 2016 (Microsoft).

## RESULTS

### Patients

Data of 28 patients were analyzed. They comprised 20 men and eight women of median age 71 years. Their histological diagnoses were squamous cell carcinoma (*n* = 18), adenocarcinoma (*n* = 9), and non‐small cell lung cancer (*n* = 1). Their disease stages were IIB (*n* = 1), IIIA (*n* = 16), IIIB (*n* = 9) and IIIC (*n* = 2). Chemotherapy regimens included weekly carboplatin + paclitaxel (*n* = 14), daily carboplatin (*n* = 13), and cisplatin + TS‐1 (*n* = 1). Programmed death ligand 1 tumor proportional scores were evaluated in 26 patients and were <1% in 10 patients, 1%–49% in seven, and ≥ 50% in nine. These data are summarized in Table 1. The correlation coefficient between volume of PTV and lung V20 was 0.01.

**TABLE 1:** Patient characteristics (*n* = 28)

Characteristics		
Age, years, median (range)		71 (36–82)
Performance status	0	16
	1	12
History of smoking	Current or former	24
	Never	4
Sex	Male	20
	Female	8
Histopathological type, n (%)	Squamous cell carcinoma	18
	Adenocarcinoma	9
	Non‐small cell lung cancer	1
T classification, n (%)	T1b	2
	T1c	4
	T2a	5
	T2b	2
	T3	3
	T4	12
N classification, n (%)	N0	3
	N1	6
	N2	14
	N3	5
Clinical stage, n (%)	IIB	1
	IIIA	16
	IIIB	9
	IIIC	2
Volume of planning target volume, ml, median (range)		200 (119–651)
Location of primary tumor	Right upper lobe	10
	Right lower lobe	7
	Left upper lobe	9
	Left lower lobe	2
Regimen of chemotherapy, n (%)	Carboplatin+paclitaxel	14
	Daily carboplatin	13
	Cisplatin+S1	1
Tumor proportion score, n (%)	<1	10
	1–49	7
	≥ 50	9
	not examined	2
Mean lung dose, Gy		11.5 (±2.2)
Lung V5, %		47.7 (±9.0)
Lung V20, %		19.2 (±4.3)

### Administration of durvalumab

The median number of cycles of durvalumab was 10. Durvalumab was discontinued in 16 of the 28 patients. Reasons for discontinuation of durvalumab comprised progression of disease in four patients, pneumonitis in six, hematological toxicities in two, diarrhea in one, hyperthyroidism in one, patient refusal in one, and anaphylactic shock in one. Administration of durvalumab was postponed in five patients due to pneumonitis.

### Incidence of RP

Over a median follow‐up of 14 months, grade 1 pneumonitis was diagnosed in 14 patients (50%), grade 2 pneumonitis in 11 (39%), and grade 3 pneumonitis in one (4%). Two patients did not develop pneumonitis. None of the patients developed grade 4 or 5 pneumonitis.

### **Cutoff values for dose‐volume parameters for predicting ≥ grade 2** **RP**

**FIGURE 3:**
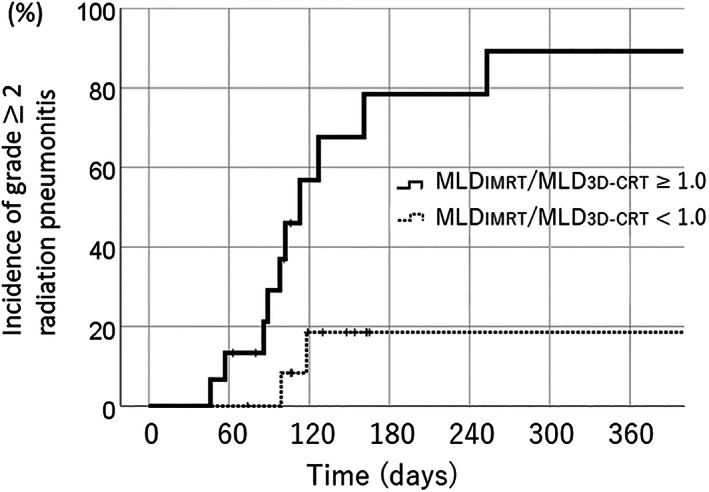
Cumulative 6‐month incidences of ≥grade 2 RP were 78.4% versus 19.5% (MLD_IMRT_/MLD_3D‐CRT,_ ≥1.0 or less) (*p* = 0.006)

Dose‐volume parameters for IMRT and simulated 3D‐CRT are shown in Table 2. ROC analysis showed that lung V5, lung V20, and MLD had relatively large AUCs (0.651, 0.607, and 0.620, respectively). Cutoff values for lung V5, lung V20, and MLD were 51.6, 21.8 and 10.5 Gy, respectively. Comparisons of the 6‐month cumulative incidence of grade 2 or greater RP between the two groups, as determined by cutoff values derived from ROC analysis, yielded the following results: 77.8% versus 39.8% (lung V5, ≥ 51.6% or less), 57.4% versus 37.5% (lung V20,  ≥10.5 Gy or less), and 100% versus 42.1% (MLD, ≥ 21.8% or less). The incidence of RP differed significantly between these two groups (*p* < 0.05). Results of these analyses are shown in Table 3. Regarding dose‐volume parameter ratios between IMRT and simulated 3D‐CRT, ROC analysis showed that MLD_IMRT_/MLD_3D‐CRT_ had the largest AUC (0.750) and cutoff value for MLD_IMRT_/MLD_3D‐CRT_ was 1.0. Cumulative 6‐month incidences of grade 2 or greater RP were 78.4% versus 19.5% (MLD_IMRT_/MLD_3D‐CRT,_ ≥1.0 or less); this difference was significant (*p* < 0.05) (Figure 3). Median MLD_IMRT_/MLD_3D‐CRT_ was 0.93 (range: 0.71–0.99) in patients whose MLD_IMRT_/MLD_3D‐CRT_ was below this cutoff value. Also, V20_IMRT_/V20_3D‐CRT_ was significantly associated with RP (*p* = 0.003). Cutoff value for V20_IMRT_/V20_3D‐CRT_ was 1.0. Cumulative 6‐month incidences of grade 2 or greater RP were 100% versus 33.5% (V20_IMRT_/V20_3D‐CRT,_ ≥1.0 or less). In this study, the cutoff value for V5_IMRT_/V5_3D‐CRT_ was 1.5; however, the association with RP was not significant (*p* = 0.087). With a simulated 3D‐CRT plan, 13 patients exceeded dose constraint for spinal cord (Dmax <50 Gy). Seven of those 13 patients satisfied cutoff value of MLD_IMRT_/MLD_3D‐CRT_. Also, eight of 13 patients satisfied cutoff value of V20_IMRT_/V20_3D‐CRT_.

**TABLE 2:** Dose‐volume parameters of IMRT and simulated 3D‐CRT

Dose‐volume parameter	IMRT	Simulated 3D‐CRT	*p*‐value
Mean lung dose, Gy	11.5 (±2.2)	11.6 (±2.8)	0.774
Lung V5, %	47.7 (±9.0)	32.7 (±7.1)	< 0.001
Lung V20, %	19.2 (±4.3)	21.7 (±5.3)	0.004
Lung V30, %	12.6 (±3.4)	18.2 (±5.0)	< 0.001
Lung V40, %	8.0 (±2.9)	15.1 (±4.4)	< 0.001
Lung V50, %	5.0 (±2.1)	10.6 (±3.4)	< 0.001
Lung V60, %	2.3 (±1.2)	1.9 (±1.7)	0.254

**TABLE 3:** Results of univariate analysis of clinical and dosimetric factors associated with incidence of ≥grade 2 pneumonitis

Variable	Cutoff value	Number of patients	Six months cumulative incidence of ≥2 RP	*p*‐value
Age (years)	≥ 63	24	51.6%	0.789
	< 63	4	100%	
Sex	Male	20	57.6%	0.635
	Female	8	41.7%	
Performance status	0	12	54.5%	0.782
	1	16	42.6%	
Stage	IIB, IIIA	17	46.5%	0.785
	IIIB, IIIC	11	60.7%	
Smoking	Never	4	25.0%	0.427
	Current or former	24	60.7%	
Brinkman index	≥ 1455	4	75%	
	< 1455	24	40.3%	
Absolute volume of PTV (ml)	≥ 151.7	23	100%	0.772
	< 151.7	5	45.1%	
Mean lung dose (Gy)	≥ 10.5	19	57.4%	0.046
	< 10.5	9	37.5%	
Lung V5 (%)	≥ 51.6	9	77.8%	0.005
	< 51.6	19	39.8%	
Lung V20 (%)	≥ 21.8	5	100%	0.009
	< 21.8	23	42.1%	
Lung V30 (%)	≥ 14.5	6	77.8%	0.111
	< 14.5	22	42.5%	
Lung V40 (%)	≥ 8.3	11	61.8%	0.191
	< 8.3	17	41.9%	
Lung V50 (%)	≥ 5.7	10	70.0%	0.061
	< 5.7	18	38.3%	
Lung V60 (%)	≥ 2.2	15	51.1%	0.602
	< 2.2	13	56.2%	
Lung V5 of contralateral lung (%)	≥ 45.3	7	57.1%	0.782
	< 45.3	21	50.6%	

### Relationship between ratio of dose‐volume parameter and PTV volume

**FIGURE 4:**
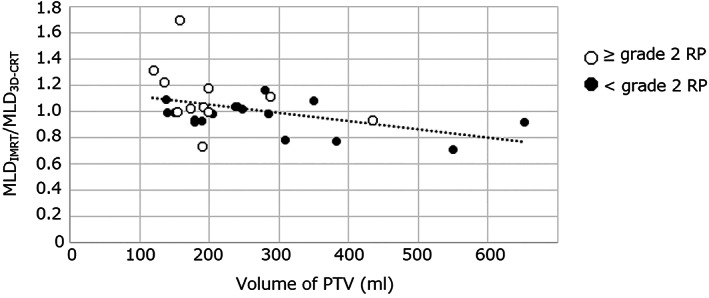
Scatter diagram of volume of PTV and MLD_IMRT_/MLD_3D‐CRT_. The dotted line represents the line of best fit, and the correlation coefficient was −0.41. The white circle represents patients with ≥grade 2 RP and the black circle represents patients with <grade 2 RP

We plotted absolute volume of PTV and ratio of dose‐volume parameters in scatter diagrams. Scatter diagrams of volume of PTV and MLD_IMRT_/MLD _3D‐CRT_ are shown in Figure 4. Approximate curves of the scatter plots showed a negative correlation between volume of PTV and V5_IMRT_/V5 _3D‐CRT_, V20_IMRT_/20 _3D‐CRT_, and MLD_IMRT_ /MLD_3D‐CRT_. Correlation coefficient were −0.30 for V5_IMRT_/V5 _3D‐CRT_, −0.42 for V20_IMRT_/20 _3D‐CRT_, and −0.41 for MLD_IMRT_ /MLD_3D‐CRT_.

## DISCUSSION

To our knowledge, this is the first study to explore means of evaluating achievement degree of lung dose optimization by IMRT followed by durvalumab for individual LA‐NSCLC patients. We first examined the dose–volume relationship between lung dose and grade 2 or greater RP and found that lung V5, lung V20, and MLD were significantly associated with grade 2 or greater RP, consistent with the findings of other studies.^8^, ^9^, ^10^ Furthermore, the ratio of dose–volume parameters between IMRT and simulated 3D‐CRT was significantly associated with grade 2 or greater RP. Clearly, the predictive ability of these cutoff values for ratios of dose‐volume parameters requires verification in a prospective study. In the meantime, this method could easily be implemented with high reproducibility in any facilities and could therefore be used as a reference for each facility to examine their own achievement degree of lung dose optimization with their own cutoff values.

In this study, the crude incidence of grade 2 or greater RP was 43%, which was consistent with reports from Asian countries.^8^, ^9^, ^10^, ^11^, ^13^, ^14^, ^15^, ^16^ We consider that our clinical data are comparable to reported results from other Asian facilities and could therefore validly be used for analyzing dose–volume relationships of RP and evaluating achievement degree of lung dose optimization.

In this study, we have shown that lung V5, lung V20, and MLD are significantly associated with grade 2 or greater RP. Tsukita et al. reported that a V5 of 58.9% is a significant predictor of grade 2 or greater RP after IMRT followed by durvalumab.^8^ Our cutoff value for lung V5 was 51.6%, which is slightly lower than that reported by Tsukita et al. However, in our opinion, this slight difference in absolute value of V5 is meaningless because these values were derived from ROC analysis and the cutoff value is easily affected by small differences in occurrence of events. However, we still believe that V5 is a useful predictor of grade 2 or greater RP and that we should minimize it as much as possible. We consider that lung V20 and MLD should also be minimized. This makes sense because, in IMRT for lung cancer, it is meaningless to prioritize only one point on the dose–volume curve; rather, it is important to reduce whole dose–volume curves by working on multiple evaluation points. This strategy is consistent with MLD being a significant factor.

In this study, we also aimed to develop methods for evaluating achievement degree of lung dose optimization in individual patients. The complexity of the optimization process and limited time in clinical situations makes it difficult to determine definite goals for lung dose optimization in individual patients. We initially created simulated 3D‐CRT plans for all patients and have described in detail how we created those plans in this study. Because beam angle, MLC margin, and prescription method were fixed in this study, there were few variations when creating such simulated 3D‐CRT plans once the target volume had been determined. According to ROC analysis, the optimal cutoff value for predicting grade 2 or greater RP was MLD_IMRT_/MLD_3D‐CRT_ of 1.0. The cumulative 6‐month incidences of grade 2 or greater RP were 78.4% versus 19.5% between the above and below cutoff value groups (*p* = 0.006). The median MLD_IMRT_/MLD_3D‐CRT_ was 0.93 (range: 0.71–0.99) among patients whose MLD_IMRT_/MLD_3D‐CRT_ were below this cutoff value. We consider that these results indicate that MLD_IMRT_/MLD_3D‐CRT_ should, at the very least, be less than 1.0, and ideally less than 0.9. We also found V20_IMRT_/V20_3D‐CRT_ to be significantly associated with RP (*p* = 0.003), whereas V5_IMRT_/V5_3D‐CRT_ was not. The cutoff value for V20_IMRT_/V20_3D‐CRT_ was also 1.0 and the median V20_IMRT_/V20_3D‐CRT_ 0.85 (0.59–0.98) among patients whose V20_IMRT_/V20_3D‐CRT_ was less than 1.0. We also consider that V20_IMRT_/V20_3D‐CRT_ should, at the very least, be less than 1.0, and ideally less than 0.85. Unlike V20 and MLD, V5_IMRT_/V5_3D‐CRT_ showed no statistical significant association with RP. Lung V5 may inevitably be higher when using IMRT than when using 3D‐CRT.^6^, ^8^, ^11^ In this study, the cutoff value for V5_IMRT_/V5_3D‐CRT_ was 1.5; however, the association with RP was not significant (*p* = 0.087). Further studies with greater numbers of patients are necessary to clarify the optimal cutoff value for V5_IMRT_/V5_3D‐CRT_.

We found that ratios of dose‐volume parameters such as V5_IMRT_/V5 _3D‐CRT_, V20_IMRT_/20 _3D‐CRT_, and MLD_IMRT_/MLD_3D‐CRT_ were negatively correlated with volume of PTV, suggesting that patients with large PTVs may benefit more from IMRT. Facilities with limited resources who must select patients for treatment with IMRT or 3D‐CRT could consider this as one objective factor for informing their decisions.

The following limitations of this study should be noted. First, the retrospective nature of the study made it difficult to eliminate biases in baseline patient characteristics. However, our patient characteristics were similar to those of other retrospective studies from Japanese facilities, as were our clinical results.^8^, ^9^, ^10^, ^11^, ^13^, ^14^, ^15^, ^16^ Second, the cutoff values in this study reflect our early experience with lung dose optimization. More efficient lung dose optimization would further lower the target value of ratio of dose‐volume parameters. Nonetheless, we believe that this method could be used as a reference to assist other facilities to determine their own cutoff values for determining achievement degree of lung dose optimization. Third, optimal dose‐volume parameter ratio would be different according to volume or location of tumor. Further studies to explore more detailed cutoff values of dose‐volume parameter ratios are warranted.

In conclusion, we found that cutoff values for lung V5, lung V20, and MLD of 51.6%, 21.8%, and 10.5 Gy significantly predict grade 2 or greater RP. Also, a cutoff value of 1.0 for both MLD_IMRT_/MLD_3D‐CRT_ and V20_IMRT_/V20_3D‐CRT_ is a significant predictor of grade 2 or greater RP. We believe that these parameters could be useful in assisting evaluation of achievement degree of lung dose optimization in IMRT for LA‐NSCLC.

## CONFLICT OF INTEREST

The authors declare no conflicts of interest.

There is no funding source to be declared.

## References

[1] National Comprehensive Cancer Network Clinical Practice Guidelines in Oncology , Non‐small cell lung cancer, Version 7. 2020. https://www.nccn.org/professionals/physician_gls/pdf/nscl.pdf. Accessed 14 May, 2022. 10.6004/jnccn.2022.0025 35545176

[2] Antonia SJ , Villegas A , Daniel D , Vicente D , Murakami S , Hui R , et al. Durvalumab after chemoradiotherapy in stage III non‐small‐cell lung cancer. N Engl J Med. 2017;377:1919–29. 28885881 10.1056/NEJMoa1709937

[3] Staffurth J . Radiotherapy Development Board. A review of the clinical evidence for intensity‐modulated radiotherapy. Clin Oncol. 2010;22:643–57. 10.1016/j.clon.2010.06.013 20673708

[4] Boyle J , Ackerson B , Gu L , Kelsey CR . Dosimetric advantages of intensity modulated radiation therapy in locally advanced lung cancer. Adv Radiat Oncol. 2017;2:6–11. 28740910 10.1016/j.adro.2016.12.006 PMC5514227

[5] Koshy M , Malik R , Spiotto M , Mahmood U , Rusthoven CG , Sher DJ . Association between intensity modulated radiotherapy and survival in patients with stage III non‐small cell lung cancer treated with chemoradiotherapy. Lung Cancer. 2017;108:222–7. 28625640 10.1016/j.lungcan.2017.04.006

[6] Chun SG , Hu C , Choy H , Komaki RU , Timmerman RD , Schild SE , et al. Impact of intensity‐modulated radiation therapy technique for locally advanced non‐small‐cell lung cancer: a secondary analysis of the NRG oncology RTOG 0617 randomized clinical trial. J Clin Oncol. 2017;35:56–62. 28034064 10.1200/JCO.2016.69.1378 PMC5455690

[7] Peng J , Pond G , Donovan E , Ellis PM , Swaminath A . A comparison of radiation techniques in patients treated with concurrent Chemoradiation for stage III non‐small cell lung cancer. Int J Radiat Oncol Biol Phys. 2020;106:985–92. 32007366 10.1016/j.ijrobp.2019.12.027

[8] Tsukita Y , Yamamoto T , Mayahara H , Hata A , Takeda Y , Nakayama H , et al. Intensity‐modulated radiation therapy with concurrent chemotherapy followed by durvalumab for stage III non‐small cell lung cancer: a multi‐center retrospective study. Radiother Oncol. 2021;160:266–72. 34023330 10.1016/j.radonc.2021.05.016

[9] Mayahara H , Uehara K , Harada A , Kitatani K , Yabuuchi T , Miyazaki S , et al. Predicting factors of symptomatic radiation pneumonitis induced by durvalumab following concurrent chemoradiotherapy in locally advanced non‐small cell lung cancer. Radiat Oncol. 2022;17:7. 35033139 10.1186/s13014-021-01979-z PMC8760798

[10] Shintani T , Kishi N , Matsuo Y , Ogura M , Mitsuyoshi T , Araki N , et al. Incidence and risk factors of symptomatic radiation pneumonitis in non‐small‐cell lung cancer patients treated with concurrent Chemoradiotherapy and consolidation Durvalumab. Clin Lung Cancer. 2021;22:401–10. 33678582 10.1016/j.cllc.2021.01.017

[11] Abe T , Iino M , Saito S , Aoshika T , Ryuno Y , Ohta T , et al. Feasibility of intensity modulated radiotherapy with involved field radiotherapy for Japanese patients with locally advanced non‐small cell lung cancer. J Radiat Res. 2021;62:894–900. 34260719 10.1093/jrr/rrab063 PMC8438249

[12] Kubo N , Kobayashi D , Iwanaga M , Matsuura M , Higuchi K , Eishima J , et al. Radiotherapy patterns of Care for Locally‐advanced non‐small Cell Lung Cancer in the pre‐ and post‐durvalumab era: a region‐wide survey in a Japanese prefecture. J Radiat Res. 2022;63:264–71. 34970980 10.1093/jrr/rrab116 PMC8944323

[13] Saito S , Abe T , Kobayashi N , Aoshika T , Ryuno Y , Igari M , et al. Incidence and dose‐volume relationship of radiation pneumonitis after concurrent chemoradiotherapy followed by durvalumab for locally advanced non‐small cell lung cancer. Clin Transl Radiat Oncol. 2020;23:85–8. 32529055 10.1016/j.ctro.2020.05.006 PMC7283100

[14] Jung HA , Noh JM , Sun JM , Lee SH , Ahn JS , Ahn MJ , et al. Real world data of durvalumab consolidation after chemoradiotherapy in stage III non‐small‐cell lung cancer. Lung Cancer. 2020;146:23–9. 32505077 10.1016/j.lungcan.2020.05.035

[15] Inoue H , Ono A , Kawabata T , Mamesaya N , Kawamura T , Kobayashi H , et al. Clinical and radiation dose‐volume factors related to pneumonitis after treatment with radiation and durvalumab in locally advanced non‐small cell lung cancer. Invest New Drugs. 2020;38:1612–7. 32128667 10.1007/s10637-020-00917-2 PMC7497668

[16] Miura Y , Mouri A , Kaira K , Yamaguchi O , Shiono A , Hashimoto K , et al. Chemoradiotherapy followed by durvalumab in patients with unresectable advanced non‐small cell lung cancer: management of adverse events. Thorac Cancer. 2020;11:1280–7. 32160383 10.1111/1759-7714.13394 PMC7180558

